# The Assessment of Cognitive Reserve: A Systematic Review of the Most Used Quantitative Measurement Methods of Cognitive Reserve for Aging

**DOI:** 10.3389/fpsyg.2022.847186

**Published:** 2022-03-31

**Authors:** Joana Nogueira, Bianca Gerardo, Isabel Santana, Mário R. Simões, Sandra Freitas

**Affiliations:** ^1^Univ Coimbra, Center for Research in Neuropsychology and Cognitive and Behavioral Intervention (CINEICC), Faculty of Psychology and Educational Sciences (FPCEUC), Coimbra, Portugal; ^2^Univ Coimbra, Psychological Assessment and Psychometrics Laboratory (PsyAssessmentLab), Faculty of Psychology and Educational Sciences (FPCEUC), Coimbra, Portugal; ^3^Geobiotec Research Centre, Department of Geosciences, University of Aveiro, Aveiro, Portugal; ^4^Univ Coimbra, Faculty of Medicine (FMUC), Coimbra, Portugal; ^5^Univ Coimbra, Center for Innovative Biomedicine and Biotechnology (CIBB), Coimbra, Portugal; ^6^Department of Neurology, Centro Hospitalar e Universitário de Coimbra (CHUC), Coimbra, Portugal

**Keywords:** cognitive reserve, assessment, measurement methods, cognitive functioning, aging

## Abstract

The cognitive reserve (CR) is widely accepted as the active ability to cope with brain damage, using preexisting cognitive and compensatory processes. The common CR proxies used are the number of formal years of education, intelligence quotient (IQ) or premorbid functioning, occupation attainment, and participation in leisure activities. More recently, it has employed the level of literacy and engagement in high-level cognitive demand of professional activities. This study aims to identify and summarize published methodologies to assess the CR quantitatively. We searched for published studies on PubMed, ScienceDirect, and Web of Science between September 2018 and September 2021. We only included those studies that characterized the CR assessment methodology. The search strategy identified 1,285 publications, of which 25 were included. Most of the instruments targeted proxies individually. The lack of a gold standard tool that incorporates all proxies and cognitive tests highlights the need to develop a more holistic battery for the quantitative assessment of CR. Further studies should focus on a quantitative methodology that includes all these proxies supported by normative data to improve the use of CR as a valid measure in clinical contexts.

## Introduction

The trajectories of typical aging are associated with a decline in several cognitive domains, whereas its expression is specific to each person. Some individuals undergo a precipitous deterioration, while others preserve their cognitive performance roughly intact, therefore presenting a successful aging (Stern et al., 2019). Besides, the heterogeneity of aging and its multiple forms of expression and cases of recovery from brain injuries, as well as the delayed emergence of symptoms in the carriers of neurodegenerative diseases, raised the hypothesis of an underlying “reserve” that mitigates the expected cognitive impairment (Stern et al., 1992, 2019). This hypothesis quickly assumed primacy in the research field, evolving for the discussion of constructs such as compensation, brain maintenance, brain reserve (BR), cognitive reserve (CR) and resulting in new approaches, methods of assessment, theoretical definitions, and exploration of their impact in human cognition (Stern et al., 1992, 2019).

From all explanatory models, BR and CR offer greater consensus. The BR refers to a passive model that states that normal cognitive functioning is sustained by neuronal subtracts that, when depleted to a critical threshold, become insufficient to maintain it (Stern, 2009, 2012; Cashman, 2011). At the same time, CR is widely accepted as the active ability to cope with brain damage, using preexisting cognitive and compensatory processes (Stern, 2009; Larson et al., 2013). During the past 3 years, there was a joint effort to develop a consensual definition, as well as research guidelines for CR, achieved through focus workgroups, consultation from expert investigators, workshops, and research studies. This framework helps design and implementation of studies in the field of CR, brain maintenance, and BR (Collaboratory on Research Definitions for Cognitive Reserve and Resilience, 2018). In the words of the NIH-collaboratory workgroup, the CR “is a property of the brain that allows for cognitive performance that is better than expected given the degree of life-course related brain changes and brain injury or disease”^1^. The flexibility and plasticity of cognitive networks, as well as the molecular and cellular mechanisms, help the brain to actively cope with age-related changes and diseases (e.g., neurodegenerative diseases; Stern, 2002; Stern et al., 2019; see Text Footnote 1). In other words, alternative networks are used to successfully complete a task or maintain normal daily performance. This is a compensatory process that reflects personal CR (Stern, 2006), and is also investigated by previous brain-imaging findings that support the cognitive performance in older adults (Davis et al., 2008). Being an active model, it is assumed that CR is influenced by various factors (e.g., life experiences, participation in stimulating environments, and education) that increase brain plasticity and resistance to cellular death (Stern, 2009; Kivimäki et al., 2015) and other age-related phenomena (e.g., synaptic and white matter changes, pathological modifications, etc.; Taylor et al., 2007; Kim et al., 2019). In fact, several studies report lower rates of cognitive decline and reduced risk of developing dementia among individuals with higher premorbid IQ, higher educational level, that engaged in leisure activities and enrolled in more cognitively demanding professional activities (Stern et al., 1994; Deary et al., 2004; Zahodne et al., 2015). CR minimizes the early expression of clinical cognitive symptomatology in brain pathology where a greater pathological load is necessary to observe the same degree of dementia symptoms in those with higher CR (Stern, 2006; Solé-Padullés et al., 2009; Mondini et al., 2016; Osone et al., 2016). Therefore, a faster decline is expected when the CR overload has been reached, with the emergence of behavioral symptoms even before a search for a possible positive biomarker’s result (Alves et al., 2012; Steffener and Stern, 2012; Jansen et al., 2015; Kivimäki et al., 2015; Mondini et al., 2016; Groot et al., 2018). In summary, the individual level of CR has been strongly associated with the maintenance of cognitive health and an active lifestyle during aging, impacting the mitigation of Alzheimer’s disease symptomatology (Clare et al., 2017; Persson et al., 2017). In cases where a better cognitive performance was observed, it is important to ensure that those differences come from longitudinal measurement results (see Text Footnote 1).

Despite its greater involvement in cognitive functioning, the objective measurement of CR remains one of the biggest challenges in the field. This is mainly due to the complexity of the CR construct that makes it difficult to operationalize. Again, on the framework of the NIH-collaboratory workgroup, we found general considerations and guidelines to deal with the CR assessment. Ideally, the CR measure should include a variable that represents the moderation of the relationship between the life course-related brain changes and the changes in cognition. The accuracy of CR measurement will be higher whenever other measures are included: (i) measures of anatomic changes (e.g., brain-imaging analysis), (ii) measures of cognition (e.g., cognitive performance and daily functioning), and (iii) CR proxy, referred to the variable that influence the relationship between (i) and (ii). This last hypothesized mechanism (CR proxy) is commonly addressed by several sociobehavioral proxies, namely the number of formal years of education, intelligence quotient (IQ), occupation attainment, and participation in leisure activities (see Text Footnote 1). Recently, it has also included the level of literacy and the engagement in high-level cognitive activities (Stern, 2009, 2012; Schoenberg et al., 2011; Larson et al., 2013; Malek-Ahmadi et al., 2018). These two last variables actively contribute to the CR, since they remain dynamic throughout life, including after the completion of formal education (Malek-Ahmadi et al., 2018; Thow et al., 2018).

Considering the difficulty in achieving adequate methods for assessing CR, some authors utilized functional imaging to analyze networks and processes likely to be involved in CR (van Loenhoud et al., 2018; Stern et al., 2018, 2019). This approach provided a better understanding of the neural mechanisms of CR, allowing the identification of relevant proxies to include in scales and questionnaires, as surrogates of the underlying brain mechanisms of CR, therefore constituting an indirect measure of this construct.

Measures of the CR vary from instruments that use one single proxy, often education (Chapko et al., 2018), to tools that include several proxies either converted into a total score or developing latent variable models (generally by principal component analysis or structural equation modeling; Conroy et al., 2010; Giogkaraki et al., 2013). The approach of using one single proxy is likely to disregard important components of a complex construct as the CR. Therefore, questionnaires that comprise multiple components seem to be the way of standardizing the CR assessment.

According to our knowledge, there is only one recent systematic review looking for properties of CR questionnaires, conducted by Kartschmit et al. (2019). They concluded about the lack of measurement quality, considering the content and structural validities, as well as responsiveness (Kartschmit et al., 2019). Similarly, Landenberger et al. (2019) also concluded about the lack of validity and the need for cross-cultural adaptation of the scales and questionnaire used to measure CR. This study aims to summarize the most used quantitative measurement methods of CR for aging, considering the post-search period of Kartschmit et al. (2019) study.

## Methodology

### Literature Search

We conducted searches for studies published between September 2018 and September 2021, considering the last systematic review by Kartschmit et al. (2019). The search terms used were “reserve” or “reserves,” “cognition,” or “brain,” “questionnaire” or “instrument” or “tool,” “cognitive reserve,” or “neuropsychological assessment.” Searches were limited to peer-reviewed publications and conducted in the following databases: Web of Science (Web of Science Core Collection, Current Content Connect MEDLINE, and SciELO), ScienceDirect, and PubMed.

### Eligibility Criteria

We only included human studies that reported at least one quantitative measure (e.g., questionnaire or tool) of CR, regardless of the presentation of its psychometric properties. We did not impose restrictions regarding the study populations and diseases, once describing the presence of ways to assess the CR. We also included any settings, i.e., clinical or research contexts. No language restrictions were made.

We excluded studies that only used sociodemographic variables to address the CR (e.g., age and educational level). Systematic reviews, meta-analyses, conferences, and workshops were also excluded, as well as articles that discuss the impact of their main goal in the CR, without describing the ways to assess it. Finally, we excluded studies related to children and adolescents (age < 18 years), but no other age restrictions were applied.

### Study Selection

**FIGURE 1:**
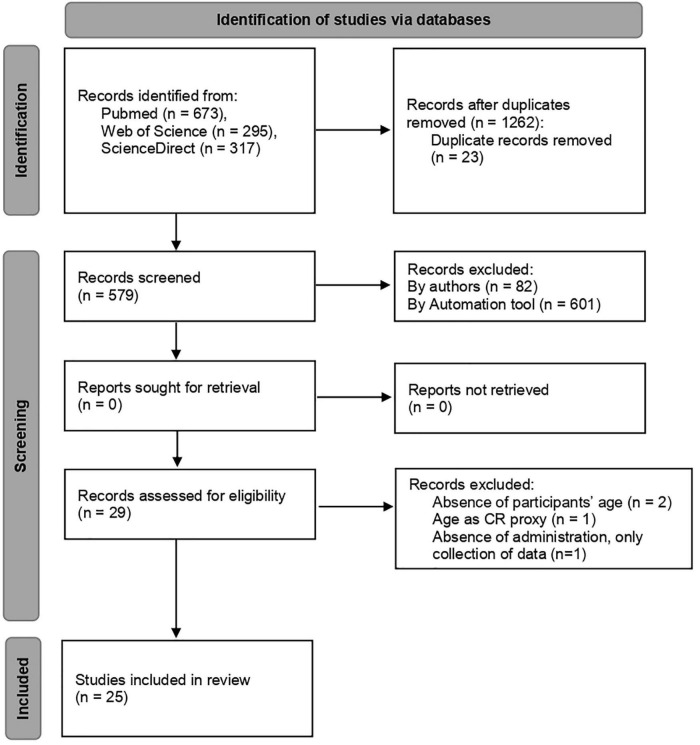
Flowchart of literature review.

Two authors (JN and BG) screened the papers and assessed them, considering the eligibility criteria. Both researchers worked independently in the abstract inspection. The discrepancies were discussed and solved by consensus. The selection process is presented in Figure 1, according to the PRISMA guidelines (Page et al., 2021).

## Results

Notably, 25 out of 579 studies screened for analysis met the inclusion criteria and were included in this study. Considering these 25 studies, the following questionnaires were the most frequently used: the Cognitive Reserve Index questionnaire (CRIq; Nucci et al., 2012), the Cognitive Reserve Questionnaire (CRQ; Rami et al., 2011), the Lifetime of Experiences Questionnaire (LEQ; Valenzuela and Sachdev, 2007), the modified version of Cognitive Reserve Scale [CRS (Leon et al., 2011), mCRS (Relander et al., 2021)], and the Cognitive Reserve Assessment Scale in Health (CRASH; Amoretti et al., 2019). Besides, four articles also included cognitive scales to assess premorbid functions.

Next, we describe the quality criteria for each way of the CR measurement: questionnaires and cognitive scales.

### Measurement Through Cognitive Reserve Questionnaires

Only 21 articles from 25 studies used questionnaires to address the CR. The major part of the questionnaires combined several sociodemographic characteristics (e.g., age and educational level) with daily living and intellectual and professional activities (refer to Supplementary Table 1).

Different instruments were used to assess CR in both healthy and clinical populations, sorted from most to least used: of 25 studies, 12 used the CRIq, 2 studies used the CRQ, 2 studies used the LEQ, 1 study used the CRASH, and 1 study used the mCRS (refer to Supplementary Table 1).

The CRIq is widely used in the field of CR assessment, with 14 cultural validation^2^ (the Turkish version presented in Supplementary Table 1) and two computations (in English and Italian versions). In this study, the CRIq was the most used questionnaire that considers validation studies and experimental approaches. Its worldwide use is an asset, since there are several validations that allow for the comparison between studies. The CRIq was composed of 20 items, which were divided into three sections, namely, CRI-education, CRI-working, and CRI-leisure time. The CRI-education was assessed based on the years of schooling. The CRI-working activity section categorizes the professional activities into five levels, i.e., from unskilled labor to management positions. If the person changes professional activity, it should be classified into a 5-year period according to the new level of employment. For the CRI-leisure times, the activities were grouped based on the frequency as follows: weekly (e.g., reading newspapers), monthly (e.g., cinema or theater), and annual frequency (e.g., exhibitions, concerts, and conferences). The CRI-leisure times also includes activities performed at a fixed frequency, such as caring for pets. Finally, the CRIq does not assess different stages of life separately.

The LEQ was the second most used questionnaire. This questionnaire is usually used in the field of aging, since it was specifically developed for participants aged ≥ 65 years. The LEQ is composed of 42 questions and includes measurements of educational, occupational, and cognitive lifestyle activities at different stages of life. Compared with CRIq, the LEQ has two main disadvantages: (1) the time of administration (approximately 30 min, whereas the CRIq takes 10–15 min) and (2) the exclusion of other age groups. However, since it was specifically developed for aging, the LEQ appears to have an advantage, which is the characterization of the previous states of functioning.

The CRQ was the third most used questionnaire in the selected studies. This questionnaire is widely used in the clinical field, especially in dementia, since it takes a few minutes to complete (Amoretti et al., 2019). It is composed of eight items that address personal schooling, training courses, parents’ schooling, professional occupation, music training, and languages proficiency. Despite its advantage of the short time of application and the scoring system based on a simple summing, the CRQ has several limitations concerning the assessment of CR, for example, the questionnaire neither evaluates different stages of life or includes a variety of leisure activities nor counts the type of courses taken or previous states of functioning.

The mCRS is a modified version of the CRS that composed of 24 items from the original version, including 20 questions about schooling and information seeking, hobbies, and social relationships. This modified version was proposed by Relander et al. (2021), who excluded the items of activities of daily living and modified some others to capture activities such as spectating sports or nature hobbies. It also includes an assessment of the frequency of those activities in different life stages. As a result, although the mCRS has the advantage of gathering information throughout life, it also has some limitations that it does not consider educational and professional attainment. The CRASH was developed specifically for severe mental illnesses, which is the major advantage of this scale for those willing to address the CR in this population. It only takes 10 min to complete the application. This scale includes the assessment of education, occupation, and intellectual and leisure activities. All domains have the same weighting in the final score, which is achieved by a formula.

Besides these questionnaires, there are four articles that used other measures (Supplementary Table 1). Dekhtyar et al. (2019) were interested in the assessment of the complexity of professional occupations, the satisfaction of social connection, and the mental, social and physical activities, besides the level of education. For this purpose, they used their own indexes, considering several stages of life span. Belleville et al. (2019) combined an adapted version of the CRQ for French with the educational level. The authors used a cognitive training program that include videogames, and so they also assessed music and game experience, multilingualism, and computer proficiency. In fact, this complimentary assessment also translates to some part of CR, since it corresponds to extra activities in addition to educational level. Darwish et al. (2018) were specifically interested in the impact of education and occupational attainment, two accepted CR factors, in the development of dementia. For this purpose, they used the International Standard Classification of Education (ISCED-11; UNESCO, 2012) and the International Standard Classification of Occupations (ISCO-08; ILO, 2012). The remaining proxies of CR were addressed using a sociodemographic questionnaire of the Dementia Research Group (DRG; Darwish et al., 2018). Finally, Szepietowska (2019) investigated the relationships between CR, the severity of depression, and cognitive functioning. Due to the lack of Polish methods to assess CR, the CRIq was used. This questionnaire contemplates a subjective assessment of life activities, formal educational level, and the nature of the occupational activity (Szepietowska, 2019).

### Measurement Through Cognitive Scales

Only four articles from 25 studies used cognitive scales to address this topic, revealing premorbid functioning as an important factor for CR. Three studies used the National Adult Reading Test (NART, Grober et al., 1991; and French adaptation of National Adult Reading Test (fNART), Mackinnon et al., 1999), and one study used the Multiple Choice Word test (MWT-B, Lehrl et al., 1995). Besides premorbid functioning, Pettigrew et al. (2019) also assessed crystallized domains, using the vocabulary scores of Wechsler Adult Intelligence Scale-Revised (WAIS-R) (Wechsler, 1981).

Pettigrew et al. (2019) used premorbid functioning (through NART), crystallized domains (through vocabulary, WAIS-R), and educational level to address the CR. These 3 measures were combined into a final score using *z*-scores and its average (Pettigrew et al., 2019). van Loenhoud et al. (2018) also used a reading test to address the CR, but they did not report others CR proxies. The MWT-B was used by Gajewski et al. (2020), which is a word meaning test that addresses crystallized intelligence. The IQ is addressed by the number of correct identification of meaningful words (Gajewski et al., 2020). They also used the years of education to complete the assessment of CR, similar to the study by Pettigrew et al. (2019).

### Cognitive Reserve Assessment Settings and Study Population

As stated earlier, our search for CR assessments comprised both clinical and research contexts. In general, the healthy population included in the selected research studies ranged between middle and old healthy aging, with an exception of van Loenhoud et al. (2020) who assessed participants aged 20–80 years. Regarding the cognitive assessments, most of the studies considered reading and vocabulary tests (i.e., NART, fNART, and vocabulary) administered to middle-aged participants. The MWT-B was applied from middle-aged to older healthy participants (Gajewski et al., 2020). Finally, the NART was also used in younger participants looking for contents that contribute to CR, considering this wide range of ages (van Loenhoud et al., 2020).

For clinical sample studies, different CR questionnaires were chosen, considering several pathologies. The CRIq was used in outpatient cohorts of multiple sclerosis (Ifantopoulou et al., 2019; Artemiadis et al., 2020), severe acquired brain injury (sABI; Bertoni et al., 2020), dementia due to Alzheimer’s disease (Montemurro et al., 2018), and substance use (Toledo-Fernández et al., 2020). The LEQ was administered to patients with the behavioral variant of frontotemporal degeneration (bvFTD; Kinney et al., 2021) and dementia (Paplikar et al., 2020). The CRQ was used in specific autoimmune encephalitis (Sola-Valls et al., 2020), and severe mental illness, as well as the CRASH, specifically developed for these cases (Amoretti et al., 2019).

## Discussion

The most accepted concept of CR is based on the theory developed by Stern (2009), but the consensus about a universal definition and the factors that should be considered for its measurement is still in discussion. However, revealing the complexity of the construct, it is assumed that the assessment should include at least one factor besides the educational level, given that schooling is an idiosyncratic feature that does not remain dynamic throughout life. For this purpose, we excluded all the papers that addressed the CR by a single proxy (e.g., educational level; Alattar et al., 2020), despite the fact that it is the easiest way to assess it in clinical populations (e.g., Alzheimer’s disease; Kwak et al., 2020).

In this study, we included studies that used quantitative measures of CR, which means questionnaires, scales, and/or cognitive tests that result in a total score that hypothetically corresponds to an individual level of CR. In contrast, we did not analyze the development of those tools or the measurement properties of the questionnaires or scales, since it was already analyzed by Kartschmit et al. (2019). We intend to summarize the quantitative methodologies used to assess the CR during the past years, discussing its instructions, scoring systems, target populations, advantages, and limitations.

We divided the articles based on those that used questionnaires and those that used cognitive scales. Regarding the measurement by questionnaires, the articles were included whenever the authors described what type of questionnaire or, preferably, the specific one that they used. We also included studies that described other quantitative ways to assess the CR, even without the use of a specific questionnaire. In those cases, the authors reported indexes or several questions as potential indicators of a personal level of CR, for example, the proficiency in languages (Narbutas et al., 2019) or social network (Dekhtyar et al., 2019). Except for the questionnaires that were specifically developed for a target population or clinical condition (e.g., CRASH for severe mental illness; Amoretti et al., 2019), most of them were used in more than one study. This means that the CR already has assessment methods that have been disseminated and validated in the scientific community for different countries. In this review, CRIq was the most used questionnaire among the reviewed articles. Considering its unrestricted life span, this questionnaire was unable to assess the specific period of time when the activities have been performed by the participants, which means it may not be answered considering the more active and functioning stage. Since the CR is intended to be a construct that is partially stable throughout life, it is important to consider the best personal time of functioning to achieve the most consistent individual level of CR. As a result, if the instructions were not provided to ensure a response related to the best functioning or more active stage of life, probably the personal level achieved does not correspond to the better persons’ level, especially in populations with memory impairment. In that case, the LEQ has the ability to specifically address earlier stages of life, since it is divided into three stages (between 13 and 30 years, from 30 to 65 years, and from 65 years). However, due to its time of administration, the LEQ is difficult to implement in clinical practice. This disadvantage of unrestricted life span is also presented in the CRQ, with the limitation of its short composition addressing a general content of a personal CR level. However, the CRQ has a 6-point Likert-type scale instead of dichotomous answers used in CRIq, which allows the measurement in terms of frequency or proficiency considering the question at hand. The same response option is used in LEQ as well as open questions. The major advantage of the CRQ in comparison with both LEQ and CRIq is the short application time for the clinical population, since it has just 8 questions and takes only 3 min (Rami et al., 2011; Sobral et al., 2014, 2015; Harris et al., 2015).

The CRIq was used in more than one disease, ranging from neurodegenerative conditions (e.g., dementia due to Alzheimer’s disease; Montemurro et al., 2018) to substance use (Toledo-Fernández et al., 2020). Recently, Stern et al. (2019) endorsed the term “cognitive resilience” as a combination of BR, CR, compensating, and brain maintenance. In clinical cases, this cognitive resilience helps to deal with aging and mitigates the impact of symptoms due to neurodegeneration (Stern et al., 2019). As a part of cognitive resilience, the use of CR assessment on clinical population is crucial to detect both people with low CR, and, consequently, with less neural resources to deal with the disease as well as those with higher levels of CR, who benefit from the mitigating effect on behavioral symptomatology. To optimize this assessment, mostly in clinical populations, it is important to obtain information about previous cognitive status, which is often not accessible. Frequently, premorbid intelligence is the indirect means to address it and plays an important role in the diagnosis of cognitive decline. Furthermore, it is also considered as a proxy of CR (Lezak et al., 2012). Therefore, several authors include neuropsychological tests to assess both premorbid and general cognitive functioning, when addressing the personal level of CR (Thow et al., 2018; Zijlmans et al., 2021). More specifically, Zijlmans et al. (2021) investigated the CR by creating a latent variable that captures variance across five cognitive tests and an MRI-inferred analysis.

However, it is important to point out the exclusive use of cognitive tests as a limitation, since it excludes the assessment of lifestyle activities that are actively involved in the CR and are usually assessed by dedicated questionnaires. Considering the complexity of the CR construct, a perfect model of assessment should include cognitive scales to address premorbid functioning and/or crystallized domains (reading and vocabulary tests, respectively), and questionnaires focusing on education, professional activities, leisure time, and social life. The approach of Thow et al. (2018) incorporating the LEQ for the assessment of life experience information and the estimated full-scale IQ (through WTAR; Thow et al., 2018) is a paradigmatic example. Likewise, the battery proposed by our group and developed specifically for the assessment of CR (Battery for the Assessment of Cognitive Reserve, BARC) has the rational of combining several questionnaires and cognitive scales computed into a single score (Nogueira et al., 2020). Ideally, the most complete paradigm of CR should include life experience information, cognitive tests, and MRI analysis. With this review, we want to emphasize that most of the instruments evaluated targeted proxies individually and between those (i) the LEQ represents a promising questionnaire to assess CR due to its extensive structure, which contains many different CR proxies, with the limitation of not addressing cognitive domains; (ii) the CRIq was the most translated CR questionnaire, which favors a further comparison between studies; (iii) the CRQ was limited in its structure but is quite simple to use in large samples or epidemiologic studies; and (iv) the two main cognitive domains considered crucial for CR assessment were the crystallized domains and premorbid functioning.

As a final statement and future perspectives concerning CR assessment, the lack of a gold standard tool, incorporating all proxies and cognitive tests, emphasizes the need to develop a more holistic battery for the quantitative assessment of CR. Further studies should focus on a quantitative methodology that includes all of these proxies and is supported by normative data to improve the use of CR as a valid measure in clinical contexts.

## Data Availability Statement

The original contributions presented in the study are included in the article/Supplementary Material, further inquiries can be directed to the corresponding author.

## Author Contributions

JN and SF contributed to the conception and design of the study. JN and BG organized the database and performed the selection and screening analysis of the articles. JN wrote the manuscript. MS, IS, and SF reviewed the methodology implemented and the results. All authors contributed to manuscript revision, read it, and approved the submitted version.

## Conflict of Interest

The authors declare that the research was conducted in the absence of any commercial or financial relationships that could be construed as a potential conflict of interest.

## Publisher’s Note

All claims expressed in this article are solely those of the authors and do not necessarily represent those of their affiliated organizations, or those of the publisher, the editors and the reviewers. Any product that may be evaluated in this article, or claim that may be made by its manufacturer, is not guaranteed or endorsed by the publisher.

## Footnotes

1
https://reserveandresilience.com/framework/

2
https://www.cognitivereserveindex.org/
