# Supplementary material for: The Assessment of Cognitive Reserve: A Systematic Review of the Most Used Quantitative Measurement Methods of Cognitive Reserve for Aging
Source: Front Psychol. 2022 Mar 31;13:847186. doi: 10.3389/fpsyg.2022.847186 (PMC9023121; doi:10.3389/fpsyg.2022.847186)
Supplement: Supplementary file 1 [file Table_1.docx]

**Table 1.** Studies included in the systematic review.

| **Study** | **Target population** | **Questionnaires or sociodemographic variables** | **Cognitive Scales** | **Assessed CR proxies** | **Life-span** | **Time administration** | **Scoring** |
| --- | --- | --- | --- | --- | --- | --- | --- |
| Amoretti et al., 2019. | Patients with severe mental illness. | [1] CRASH (Cognitive Reserve Assessment Scale in Health);  [2] CRQ (Cognitive Reserve Questionnaire). | Without information. | [1] Education, occupation, and intellectual and leisure activities.[2] CRQ: 8 items of intellectual activities (e.g.: schooling, musical training, etc.) | [1] Childhood and adolescence; adulthood and the previous year. [2] Unrestricted. | [1] 10 minutes.  [2] 2 minutes. | [1] CRASH: Formula with the same weighting of each domain in the final score.  [2] Simple summing of the 8 items. |
| Artemiadisa et al., 2020. | Multiple Sclerosis outpatients. | Cognitive Reserve Index (CRIq: [1] CRI-Education, [2] CRI-Working Activities, [3] CRI-Leisure Time Activities). | Without information. | [1] Education (years) and other educational instruction;[2] 5 categories of working activities;[3] Frequency of leisure activities. | Unrestricted (i.e., experiences that a person has acquired throughout theirlife). | 10-15 minutes. | Combination of the 3 sub-scores, adjusted for age and gender. |
| Belleville et al., 2019. | Participants aged 60–85 years with memory complaints (from COMPASS-ND study cohort. | [1] Education Level; [2] Cognitive Reserve; Questionnaire (modified); [3] Music Experience Questionnaire; [4] Language Experience and Proficiency Questionnaire (LEAP). | Without information. | [1] years of education; [2] modified version of CRQ (usually include the assessment of activities (e.g.: schooling, musical training, etc.); [3] experience with music; [4] multilingualism; | Without information. | Without information. | Without information. |

**Table 1.** (Continued)

| **Study** | **Target population** | **Questionnaires or sociodemographic variables** | **Cognitive Scales** | **Assessed CR proxies** | **Life-span** | **Time administration** | **Scoring** |
| --- | --- | --- | --- | --- | --- | --- | --- |
| Belleville et al., 2021. | Older participants from the Quebec Consortium. | Cognitive Reserve Index (CRIq: [1] CRI-Education, [2] CRI-Working Activities, [3] CRI-Leisure Time Activities). | Without information. | [1] Education (years) and other educational instruction; [2] 5 categories of working activities; [3] Frequency of leisure activities. | Unrestricted (i.e., experiences that a person has acquired throughout their life). | 10-15 minutes. | Combination of the 3 sub-scores, adjusted for age and gender. |
| Bertoni et al., 2020. | Patients with severe acquired brain injury (sABI). | Cognitive Reserve Index (CRIq: [1] CRI-Education, [2] CRI-Working Activities, [3] CRI-Leisure Time Activities). | Without information. | [1] Education (years) and other educational instruction; [2] 5 categories of working activities; [3] Frequency of leisure activities. | Unrestricted (i.e., experiences that a person has acquired throughout their life). | 10-15 minutes. | Combination of the 3 sub-scores, adjusted for age and gender. |
| Çebi and Kulce, 2021. | Participants from 18 to 80 years old. | Cognitive Reserve Index (CRIq: [1] CRI-Education, [2] CRI-Working Activities, [3] CRI-Leisure Time Activities). | Not applicable. | [1] Education (years) and other educational instruction; [2] 5 categories of working activities; [3] Frequency of leisure activities. | Unrestricted (i.e., experiences that a person has acquired throughout their life). | 10-15 minutes. | Combination of the 3 sub-scores, adjusted for age and gender. |

**Table 1.** (Continued)

| **Study** | **Target population** | **Questionnaires or sociodemographic variables** | **Cognitive Scales** | **Assessed CR proxies** | **Life-span** | **Time administration** | **Scoring** |
| --- | --- | --- | --- | --- | --- | --- | --- |
| Darwish et al., 2018. | Patients with dementia and aged 65 years old and older. | [1] 10 Items from sociodemographic questionnaire of Dementia Research Group (DRG); [2] International Standard Classification of Education (ISCED-11); [3] International Standard Classification of Occupations (ISCO-08); [4] Metabolic Equivalent of Task -minute (MET-min). | Without information. | [2] Level of education; [3] The complexity of highest attained occupation, current employment status; [4] Physical activity. (*There were no specific questionnaires to assess the social and leisure time activities, although the frequency of physical activity was included in social activity*.) | Without information. | Without information. | All the proxies were scored independently one from each other. There was no total score for CR. |
| Dekhtyar et al., 2019. | Participants aged 60 years old and older. | [1] Years of education (i.e., classification);[2] Substantive Work Complexity; [3] Social Network Index; [4] Leisure activities Index. | Without information. | [1] Levels of education; [2] Professional occupations; [3] Satisfaction with social connection;[4] Mental, social and physical activities. | [1] Early Life;  [2] Midlife;  [3] Late Life;  [4] Late Life. | Without information. | [1] Number of years of education;  [2] Continuous score;  [3] Continuous score;  [4] Simple summing of the total score of 3 domains. |
| Gajewski et al., 2020. | Young, middle-aged and healthy old participants. | Years of education. | Multiple-choice word-test (MWT-B). | Education levels (e.g.: primary, high school, etc.) and IQ (by crystallized intelligence). | Not applicable. | Without information. | Education was measured in levels and IQ by the scoring system of MWT-B. |

**Table 1.** (Continued)

| **Study** | **Target population** | **Questionnaires or sociodemographic variables** | **Cognitive Scales** | **Assessed CR proxies** | **Life-span** | **Time administration** | **Scoring** |
| --- | --- | --- | --- | --- | --- | --- | --- |
| Golja et al. 2020. | Participants aged 65 years old and older. | Cognitive Reserve Index (CRIq: [1] CRI-Education, [2] CRI-Working Activities, [3] CRI-Leisure Time Activities). | Without information. | [1] Education (years) and other educational instruction; [2] 5 categories of working activities; [3] Frequency of leisure activities. | Unrestricted (i.e., experiences that a person has acquired throughout their life). | 10-15 minutes. | Combination of the 3 sub-scores, adjusted for age. |
| Ifantopoulou et al., 2019. | Multiple Sclerosis outpatients. | Cognitive Reserve Index (CRIq: [1] CRI-Education, [2] CRI-Working Activities, [3] CRI-Leisure Time Activities). | Without information. | [1] Education (years) and other educational instruction; [2] 5 categories of working activities; [3] Frequency of leisure activities. | Unrestricted (i.e., experiences that a person has acquired throughout their life). | 10-15 minutes. | Combination of the 3 sub-scores, adjusted for age. |
| Kinney et al., 2021. | Behavioral variant fronto-temporal degeneration (bvFTD) patients. | Lifetime of experiences questionnaire (LEQ). | Without information. | [1] Education (years); [2] Occupational; [3] Social/leisure experiences. | [1] young adulthood, [2] mid-life and [3] late-life. | Without information. | Total LEQ score: combination of the three life stage scores.  *Note: Responded by the informant/caregiver.* |

**Table 1.** (Continued)

| **Study** | **Target population** | **Questionnaires or sociodemographic variables** | **Cognitive Scales** | **Assessed CR proxies** | **Life-span** | **Time administration** | **Scoring** |
| --- | --- | --- | --- | --- | --- | --- | --- |
| Martincevic and Vranic, 2021. | Older adults participants aged between 60 and 80. | Cognitive Reserve Index (CRIq: [1] CRI-Education, [2] CRI-Working Activities, [3] CRI-Leisure Time Activities). | Without information. | [1] Education (years) and other educational instruction; [2] 5 categories of working activities; [3] Frequency of leisure activities. | Unrestricted (i.e., experiences that a person has acquired throughout their life). | 10-15 minutes. | Combination of the 3 sub-scores, adjusted for age and gender. |
| Montemurro et al., 2018. | Elderly patients with major or mild neurocognitive disorder due to Alzheimer’s disease (59 and 86 years). | Cognitive Reserve Index (CRIq: [1] CRI-Education, [2] CRI-Working Activities, [3] CRI-Leisure Time Activities). | Without information. | [1] Education (years) and other educational instruction;[2] 5 categories of working activities;[3] Frequency of leisure activities. | Unrestricted (i.e., experiences that a person has acquired throughout their life). | 10-15 minutes. | Combination of the 3 sub-scores, adjusted for age and gender. |
| Montemurro et al., 2019. | Participants aged between 65 and 96 years. | Cognitive Reserve Index (CRIq: [1] CRI-Education, [2] CRI-Working Activities, [3] CRI-Leisure Time Activities). | Without information. | [1] Education (years) and other educational instruction; [2] 5 categories of working activities; [3] Frequency of leisure activities. | Unrestricted (i.e., experiences that a person has acquired throughout their life). | 10-15 minutes. | Combination of the 3 sub-scores, adjusted for age and gender. |

**Table 1.** (Continued)

| **Study** | **Target population** | **Questionnaires or sociodemographic variables** | **Cognitive Scales** | **Assessed CR proxies** | **Life-span** | **Time administration** | **Scoring** |
| --- | --- | --- | --- | --- | --- | --- | --- |
| Narbutas et al., 2019. | Healthy late middle-aged participants. | Without information. | French version National Adult Reading Test (fNART). | [1] Educational level; [2] Occupational demands; [3] Physical activities; [4] Leisure activities across the lifespan. | Only the leisure activities are assessed considering the lifespan. | Without information. | The IQ was scored by the fNART’ system. |
| Ozakbas et al., 2021. | Middle-aged healthy participants | Cognitive Reserve Index (CRIq: [1] CRI-Education, [2] CRI-Working Activities, [3] CRI-Leisure Time Activities). | Without information. | [1] Education (years) and other educational instruction; [2] 5 categories of working activities; [3] Frequency of leisure activities. | Unrestricted (i.e., experiences that a person has acquired throughout their life). | 10-15 minutes. | Combination of the 3 sub-scores, adjusted for age and gender. |
| Paplikar et al., 2020. | Healthy elderly participants and dementia patients. | Lifetime of Experiences Questionnaire (LEQ). | Without information. | [1] Education (years); [2] Occupational; [3] Social/leisure experiences. | [1] young adulthood, [2] mid-life and [3] late-life. | 30–35 minutes. | Total LEQ score: combination of the three life stage scores.  *Note: Responded by the informant/caregiver.* |
| Pettigrew et al., 2019. | Participants middle-aged (M=56.6 years). | [1] Level of education. | [2] National Adult Reading Test (NART).  [3] WAIS-R vocabulary. | [1] Years of education;  [2] Premorbid IQ;  [3] Crystallized intelligence. | Not applicable. | Without information. | Total score composed by the three measures through averaged z-scores. |

**Table 1.** (Continued)

| **Study** | **Target population** | **Questionnaires or sociodemographic variables** | **Cognitive Scales** | **Assessed CR proxies** | **Life-span** | **Time administration** | **Scoring** |
| --- | --- | --- | --- | --- | --- | --- | --- |
| Relander et al., 2021. | Neurologically healthy adults aged 26–78. | Modified Cognitive Reserve Scale (mCRS). | Without information. | [1] Studying and information seeking;  [2] Hobbies;  [3] Social relationships. | Young adulthood (18–35 years), adulthood (36–64 years) and late adulthood (65 years). | Without information. | Simple summing of the averaged item scores. |
| Sola-Valls et al., 2020. | Healthy controls and patients with anti–leucine-rich, glioma-inactivated 1 (LGI1) encephalitis. | Cognitive reserve questionnaire (CRQ). | Without information. | Cognitive Reserve Questionnaire addressed: [1] educational level and training courses; [2] educational level of parents; [3] work occupation performed throughout life; [4] musical education; [5] ability to speak several languages. | Without information. | Without information. | Simple summing of the items. |
| Szepietowska, 2019. | Participants aged from 44 to 84 years. | Cognitive Reserve Index (CRIq: [1] CRI-Education, [2] CRI-Working Activities, [3] CRI-Leisure Time Activities). | Without information. | [1] Education (years) and other educational instruction; [2] 5 categories of working activities; [3] Frequency of leisure activities. | Unrestricted (i.e., experiences that a person has acquired throughout their life). | 10-15 minutes. | Combination of the 3 sub-scores, adjusted for age and gender. |

**Table 1.** (Continued)

| **Study** | **Target population** | **Questionnaires or sociodemographic variables** | **Cognitive Scales** | **Assessed CR proxies** | **Life-span** | **Time administration** | **Scoring** |
| --- | --- | --- | --- | --- | --- | --- | --- |
| Szepietowska, 2020. | Participants aged 40 years old and older. | [1]CR-interview;  [2]CR-interview/education;  [3]CR-interview/ education/occupation. | Without information. | [1] Subjective assessment of life activities; [2] formal education level; [3] formal nature of occupational activity. | Without information. | Without information. | [1] CR-interview was determined based on Cognitive Reserve Index questionnaire; [2] The total of CR-interview and the level of formal education; [3]CR-interview_education_occupation comprised CR-interview/education and the current occupational status. |
| Toledo-Fernández et al., 2020. | Male participants in residential treatment (between 18 and 60 years old). | Cognitive Reserve Index (CRIq: [1] CRI-Education, [2] CRI-Working Activities, [3] CRI-Leisure Time Activities). | Without information. | [1] Education (years) and other educational instruction; [2] 5 categories of working activities; [3] Frequency of leisure activities. | Unrestricted (i.e., experiences that a person has acquired throughout their life). | 10-15 minutes. | Combination of the 3 sub-scores, adjusted for age and gender. |
| van Loenhoud et al., 2018. | Participants between 20 and 80 years old. | Without information. | American National Adult Reading Test (NART). | Education and premorbid IQ levels. | Not applicable. | Without information. | Education was measured in years and premorbid IQ by the scoring system of NART. |

References:

Belleville, S., Mellah, S., Cloutier, S., Dang-Vu, T. T., Duchesne, S., Maltezos, S., et al. (2021). Neural correlates of resilience to the

effects of hippocampal atrophy on memory. NeuroImage: Clin. 29:102526. doi: 10.1016/j.nicl.2020.12526

Çebi, M., and Kulce, S. N. (2021). The Turkish translation study of the Cognitive Reserve Index Questionnaire (CRIq). Appl. Neuropsychol.

Adult. Online ahead of print. doi: 10.1080/23279095.2021.1896519

Golja, K., Daugherty, A. M., and Kavcic, V. (2020). Cognitive reserve and depression predict subjective reports of successful aging.

Arch. Gerontol. Geriatr. 90:104137. doi: 10.1016/j.archger.2020.104137

Martinˇcevi´c, M., and Vrani´c, A. (2021). The higher, the better: cognitive reserve contributes to lifestyle activities in older age. Appl.

Neuropsychol. Adult. Online ahead of print. doi: 10.1080/23279095.2021.1950154

Montemurro, S., Mondini, S., Crovace, C., and Jarema, G. (2019). Cognitive reserve and its effect in older adults on retrieval of proper

names, logo names and common nouns. Front. Commun. 4:14. doi: 10.3389/fcomm.2019.00014

Ozakbas, S., Yigit, P., Akyuz, Z., Sagici, O., Abasiyanik, Z., Ozdogar, A. T., et al. (2021). Validity and reliability of “Cognitive Reserve

Index Questionnaire” for the Turkish Population. Multiple Sclerosis Related Disord. 50:102817. doi: 10.1016/j.msard.2021.102817

Szepietowska, E. (2020). Mediatory effect of depression in the relations between cognitive reserve and cognitive abilities. does a

CR index matter?. Health Psychol. Rep. 7, 200–212.
